# Molecular characterization of some multidrug resistant *Candida Auris* in egypt

**DOI:** 10.1038/s41598-025-88656-3

**Published:** 2025-02-10

**Authors:** Sara H. Ahmed, Iman M. A. El-Kholy, Adel A. El-Mehalawy, Eman M. Mahmoud, Nadia A. Elkady

**Affiliations:** 1 Microbiology Department, Faculty of Science, Ain Shams University, Cairo, Egypt; 2 Clinical Pathology Department, Ain Shams University Specialized Hospital, Ain Shams University, Cairo, Egypt; 3 Microbiology Department, Abou Al-Azayem Hospital, Cairo, Egypt

**Keywords:** *Candida auris*, *ERG*, *FKS*, Gene resistance, Multidrug resistance, Azoles, Echinocandins., Clinical microbiology, Fungi, Microbial genetics, Microbiology, Health care, Medical research

## Abstract

*Candida auris* is an emerging multidrug-resistant yeast that causes healthcare-associated and deep-seated infections. Notably, the emergence of this yeast is alarming as it exhibits resistance to azoles, echinocandins, and amphotericin B, which may lead to clinical treatment failure in patients. This study aims to identify and characterize the genetic determinants of antifungal resistance in *C. auris* among some local clinical isolates to contribute for understanding the molecular epidemiology of *C. auris* in Egypt. Four test strains were identified based on the ribosomal internal transcribed spacer (ITS) region sequence and phylogenetic analysis. Antifungal susceptibility was determined using the VITEK 2 system. Molecular analysis of *ERG11*,* ERG3*,* FKS1*, and *FKS2* was used to identify mutations associated with antifungal resistance. The four test strains were identified as *C. auris*. Evolutionary analysis was conducted, and sequences of ITS regions were submitted to GenBank. The mutations Y132F in *ERG11* and F635Y in *FKS2* were identified, which are known to confer resistance to azoles and echinocandins, respectively. The emergence of *C. auris* in Egypt represents a public health concern. Hospitals should implement strict infection control measures to prevent its spread. Effective treatment guidelines and ongoing monitoring of antifungal resistance are essential to combat this emerging pathogen.

**Supplementary Information:**

The online version contains supplementary material available at 10.1038/s41598-025-88656-3.

## Background

*C. auris *was identified as a global health threat worldwide^1^. In 2019, The Centers for Disease Control and Prevention (CDC) listed *C. auris *as the only fungal pathogen among the five most urgent threats in its Antibiotic Resistance Threats Report^2^. Recently, *C. auris *was ranked second in the critical group in the Fungal Pathogens Priority List released by The World Health Organization (WHO)^3^. This is due to its global emergence, persistent inter- and intra-hospital transmissions, proclivity to colonize both patients and abiotic surfaces, high outbreak potential, challenging diagnosis, high mortality rates, thermal resistance, partial resistance to commonly used disinfectants; and most importantly intrinsic and acquired resistance to antifungal treatment^4,5^. *C. auris *can displaymultidrug resistance, with alarming resistance to first-line antifungal drugs^6^. This poses a significant risk of clinical treatment failure in patients^7^

Whole genome sequencing and phylogenetic studies distributed *C. auris *into four main groups by region: South Asian (clade I), East Asian (clade II), African (clade III), and South American (clade IV)^8,9^. A smaller group was identified as clade V based on an isolate recovered from an ear discharge of a patient from Iran. Clades I to IV are considered the most predominant clades with a wide global distribution, while Clade V showed less prevalence^6,9,10^. In 2024, researchers in Bangladesh conducted whole-genome sequencing (WGS) on *C. auris* isolates and identified a genetically distinct clade, designated as Clade VI. This clade exhibited unique genetic markers, setting it apart from the previously known clades I through V. Similar study from Singapore reported the detection of C. *auris *isolates that were genetically distinct from all known clades, further supporting the existence of Clade VI^8^. Although resistance levels vary considerably between clades, clinical isolates of *C. auris *infections have interestingly displayed clinical drug resistance to all known antifungal classes used systemically: azoles (e.g. fluconazole), polyenes (e.g. amphotericin B) and echinocandins (e.g. caspofungin)^9,11^. It has been reported that approximately 87–100% of *C. auris *isolates have shown resistance to fluconazole, up to 35% to amphotericin B, and 0–8% to echinocandins^4,12^. Moreover, *C. auris *can develop resistance to individual drugs and combinations of drugs from the three primary antifungal classes^13^.

Several studies explained the possible mechanisms behind the ability of *C. auris* to resist antifungal drugs. Point mutation of the ergosterol-biosynthesis genes *ERG11* and *EGR6* was a mechanism of fluconazole resistance repeatedly identified in *C. auris*^14,15^. Moreover, mutations in *FKS1*, a gene coding for 1,3 -β-d-glucan synthases, are the major cause of resistance to echinocandins^14,16^. Mutations in ergosterol-biosynthesis genes *ERG2*, *ERG3*, *ERG5*, *ERG6*, and *ERG11* reduce the susceptibility of *C. auris *to amphotericin B^11,14^.

Given the limited information available about *C. auris *in Egypt, insights from molecular characterization of resistance genes of local clinical isolates can help understand resistance mechanisms, guide clinical treatment, develop targeted therapies, monitor and surveillance, and contribute to global health efforts.

## Methods

### Test strains

This study aims to investigate the antimicrobial resistance pattern of Egyptian *Candida auris* isolates. For this purpose, four strains were selected from previously identified *C. auris *isolates (a surveillance study under publication). The isolates were recovered from clinical specimens collected from patients admitted to Ain Shams University Specialized Hospital (Cairo, Egypt). Specimens included blood, urine, sputum, skin swab, and bronchoalveolar lavage (BAL). They were initially incubated at 42 ^o^C for 48 h and then identified by Vitek 2 System (bioMérieux, France)^3,17^. The identification was further confirmed by Matrix-assisted laser desorption ionization-time of flight (MALDI-TOF) mass spectrometry (LT2 plus, SAI, UK)^17^. The isolates were subsequently tested for antifungal susceptibility to fluconazole, voriconazole, amphotericin B, flucytosine, ketoconazole, itraconazole, caspofungin, and micafungin. These tests were conducted following the Clinical Laboratory Standards Institute (CLSI) broth microdilution (BMD) protocol CLSI M27-A3^18^ and utilizing the Vitek 2 system (bioMérieux, France). As CLSI and the European Committee on Antimicrobial Susceptibility Testing (EUCAST) have not yet defined all antifungals breakpoints for *C. auris*, provisional breakpoints proposed by the U.S. Centers for Disease Control and Prevention (CDC) has been used. These include fluconazole at 32 mg/L, amphotericin B at 2 mg/L, micafungin at 4 mg/L, and caspofungin at 2 mg/L. Additionally, tentative breakpoints for other antifungal agents such as voriconazole and flucytosine (2 mg/L and 64 mg/L, respectively) were used as suggested in previously published studies^19,20^. Since the breakpoints for posaconazole, itraconazole and ketoconazole are not defined, breakpoints of multidrug resistant *Candida tropicalis & Candida parapsilosis* were used based on CLSI guidelines. The test strains were selected based on their resistance to different classes of antifungal agents. They were coded INSF1, INSF3, INSF5, and INSF6.“The study was approved by Ain Shams University Specialized Hospital and all methods were performed in accordance with the relevant guidelines and regulations.”

## Identification by sequences of the internal transcribed spacer region of the ribosomal RNA (ITS rRNA)

Total genomic DNA from isolates INSF1, INSF3, INSF5, and INSF6 was extracted as per the manufacturer’s instructions using Qiagen’s Yeast/Bact. Kit (Gentra Pure gene Yeast/Bact. Kit, Qiagen, Germany). The *ITS rRNA* gene (450 bp) of all the strains was amplified using *ITS rRNA *F (5’- TCCGTAGGTGAACCTGCGG-3’) and *ITS rRNA *R (5’- TCC TCC GCT TAT TGA TAT GC-3’) primers^21^, at annealing temperature 56 °C using absolute master mix (Emerald Amp GT PCR master mix (Takara, Japan) Code No. RR310Akit) in Professional thermocycler (Biometra, Germany) for 35 cycles. PCR products were purified using a QIA quick PCR Product extraction kit (Qiagen, Valencia) after visualizing the product using 1.5% agarose gel. BigDye™ Terminator v3.1 Cycle Sequencing Kit (Perkin Elmer Applied Biosystems, Foster City, CA, USA) was used for the sequence reaction and then it was purified using a Centrisep spin column purification system. DNA sequences were obtained by Applied Biosystems 3130 genetic analyzer (HITACHI, Japan), and a BLAST^®^(Basic Local Alignment Search Tool) analysis was initially performed to establish sequence identity to GenBank accessions^22^. Phylogenetic analyses were done using maximum likelihood, neighbor-joining, and maximum parsimony in MEGA6^23^.

## Detection of resistance genes

Primers for the *ERG3*,* ERG11*,* FKS1*, and *FKS2 *genes were purified and subjected for sanger sequencing^6,13,24^. All the primers were provided by Metabion, Germany. The genes were amplified by absolute master mix (Emerald Amp GT PCR master mix (Takara, Japan) in a Professional thermocycler (Biometra, Germany) for 35 cycles. Amplicons were visualized using 1.5% agarose gel and documented. The mutation was detected by using Mega (version 6) software by comparing with *C. auris* isolate AR0382 for *ERG11* (GenBank accession MK294628) and *C. auris* strain B11220 for *FKS1*,* FKS2*, and *ERG3* (GenBank accession CP043531). The primers’ sequence, orientation, annealing temperatures, and amplicon size are listed in Table 1.

**Table 1:** Primers used in the detection of resistance genes.

Gene	Primer and orientation	Annealing temp. (℃)	Product size (bp)	Ref
*ERG3*	F (5’ TCAACGGATTCTCCAAGC 3’)	56	232	^13^
	R (5’TGGAACCATCCGTCAACTG 3’)			
*ERG11*	F (5’GTGGGCTCTGCTGTTGTTTA 3’)	52	330	^6^
	R (5’CAAAACTTCCTCTTGGATTCTG 3’)			
*FKS1*	F (5’ATTTCAGAAGGAACCTGG 3’)	56	723	^13^
	R (5’CGTTCCATTCGCTTATTC 3’)			
*FKS2*	F (5’AAATGGAAGGGTTGCACTTG 3’)	52	377	^24^
	R (5’ TCCAAGGCGTCCAGATAGAT 3’)			

## Results

The test organisms were collected from patients with different comorbidity diseases. They were all capable of growth on Sabouraud’s dextrose agar at 42 ℃. They were identified as *C. auris* by MALDI-TOF with confidence values 0.8–1 on BactoScreen-ID version 2.4. Upon screening for antifungal resistance, it was found that all the test strains were resistant to first-generation azoles (fluconazole, itraconazole, and ketoconazole), echinocandins (micafungin and caspofungin), and flucytosine. However, they were susceptible to the second generation of azoles (posaconazole and voriconazole) and the polyene antifungal amphotericin B. The underlying condition, type of specimen, and antifungal susceptibility are listed in Table 2.

**Table 2:** The underlying condition, type of specimen, and antifungal susceptibility.

Isolate	Underlying Condition	Clinical Manifestation of *C. auris*	Specimen	Antifungal Susceptibility (ug/ml)								
				Flc	Itra	Keto	Fct	Mcf	Caspo	Pos	Vori	AB
**INSF 1** Acc. No: OR976106	Post covid19 (DM)	Sepsis	Blood	32 (R)	≥ 16 (R)	32 (R)	≥ 64 (R)	≥ 8 (R)	≥ 8 (R)	1 (S)	0.25 (S)	1 (S)
**INSF 3** Acc. No: OR976107	Post covid19 (DM)	UTI	Urine	32 (R)	≥ 16 (R)	32 (R)	≥ 64 (R)	≥ 8 (R)	≥ 8 (R)	1 (S)	1 (S)	1 (S)
**INSF 5** Acc. No: OR976108	Post covid19 (DM)	Sepsis	Blood	32 (R)	≥ 16 (R)	32 (R)	≥ 64 (R)	≥ 8 (R)	≥ 8 (R)	0.25 (S)	1 (S)	1 (S)
**INSF 6** Acc. No: OR976109	Liver transplant	Pneumonia	BAL	32 (R)	≥ 16 (R)	32 (R)	≥ 64 (R)	≥ 8 (R)	≥ 8 (R)	0.25 (S)	0.25 (S)	1 (S)

DM: Diabetes Mellitus, Flc: Fluconazole, Itra: Itraconazole, Keto: Ketoconazole, Fct: Flucytosine, Mcf: Micafungin, Caspo: Caspofungin, Pos: Posaconazole, Vori: Voriconazole, AB: Amphotericin B, S: sensitive, R: resistant.

## Molecular identification

**Fig. 1:**
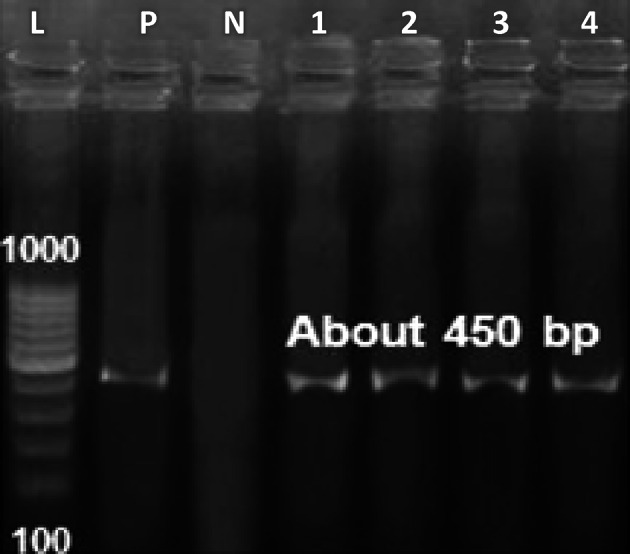
Gel electrophoresis of the amplified ITS sequences. **L**: DNA Ladder (100 bp and 1000 bp); **P**: positive control; **N**: negative control; **1**: INSF1; **2**: INSF3; **3**: INSF5; and **4**: INSF6.

**Fig. 2:**
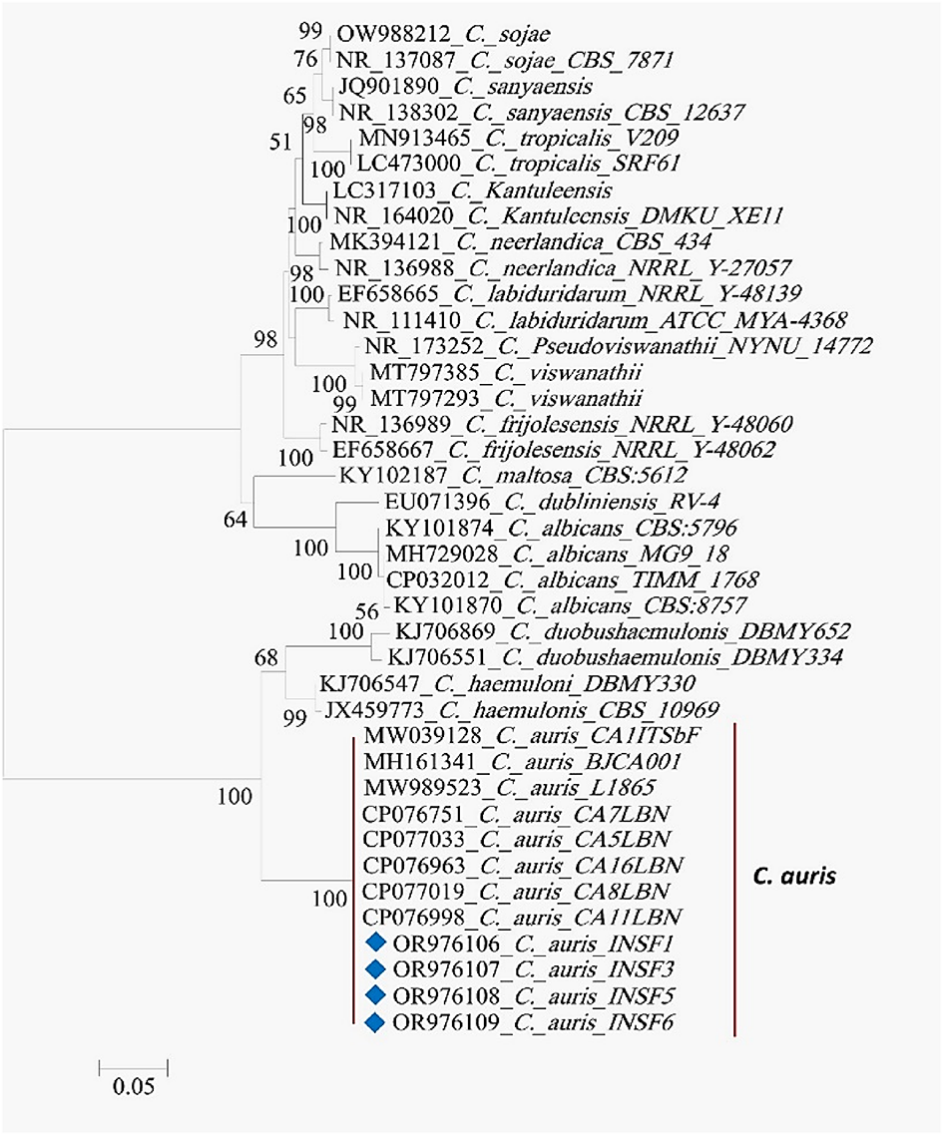
Phylogenetic tree analysis locates *C. auris* as a distinct group, indicating that it is genetically and evolutionarily distinct from other species.

The molecular identification of the test strains was conducted by comparing the ITS1-5.8 S-ITS2 ribosomal RNA region sequences obtained from the isolates (Fig. 1) to reference strains in the GenBank database using a BLAST homology search. This analysis unequivocally identified the recovered strains as *C. auris*. The sequences of the 18 S rRNA ITS region for the test strains were subsequently submitted to GenBank (accession numbers were provided in Table 1). A phylogenetic analysis was performed using the neighbor-joining method based on the ITS region sequence data to investigate evolutionary relationships among the isolates. A consensus tree was constructed from 500 bootstrap replicates, including 58 nucleotide sequences with a total of 333 positions in the final dataset. The resulting phylogenetic tree is depicted in Fig. 2, illustrating the clustering of INSF 1, 3, 5, and 6 isolates together with other *C. auris* strains.

### Detection of resistance genes

**Fig. 3:**
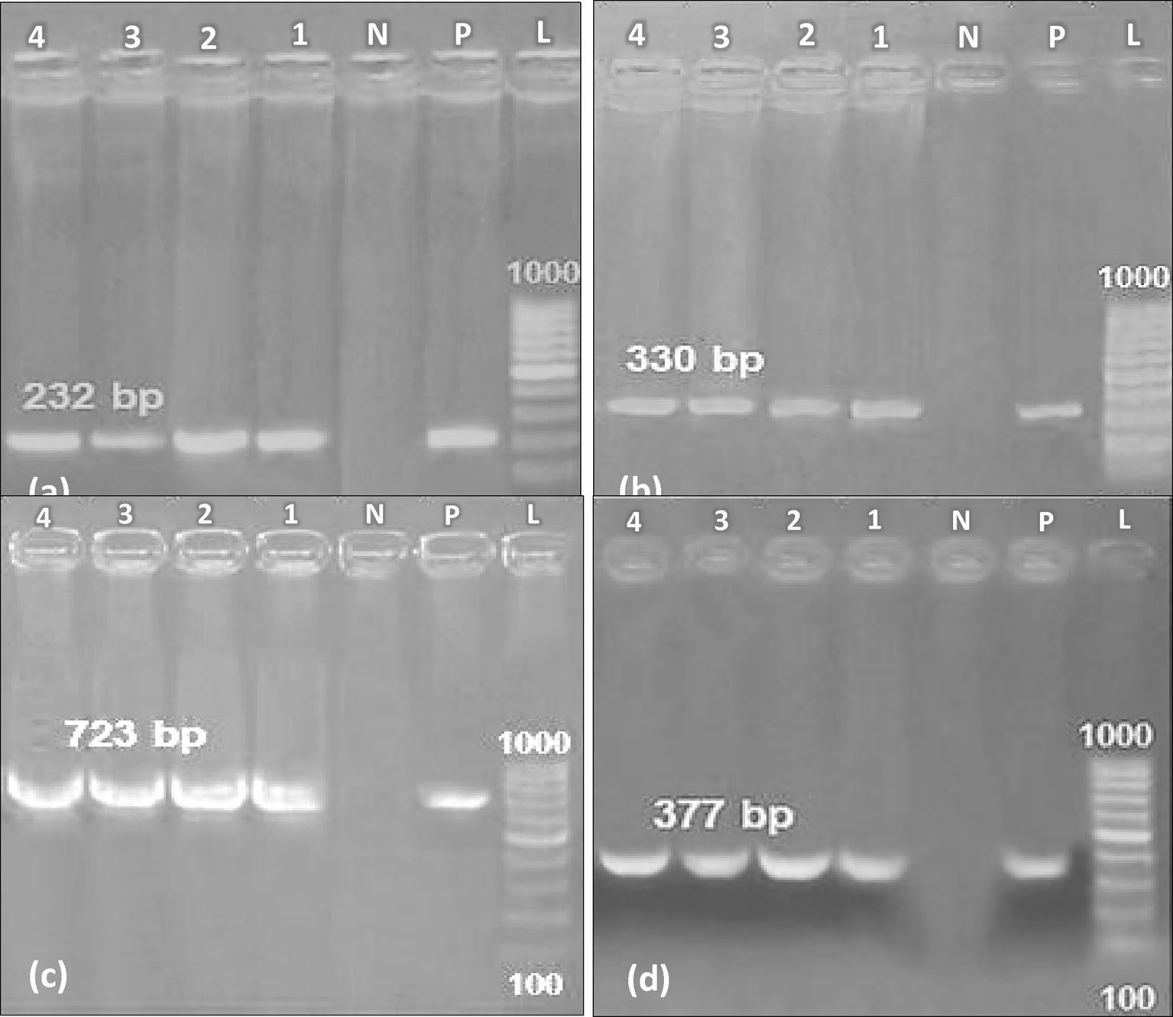
Gel electrophoresis of the amplified genes: **(a) ***ERG3*; **(b)** *ERG11*; **(c)** *FKS1*; **(d)** *FKS2*; **L**: DNA Ladder (100 bp and 1000 bp); **P**: positive control; **N**: negative control; **1**: INSF1; **2**: INSF3; **3**: INSF5; and **4**: INSF6.

All isolates were positive for the *ERG11* (~ 330 bp), *ERG3* (~ 232 bp), *FKS1* (~ 723 bp), and *FKS2* (~ 377 bp) genes (Fig. 3). Mutations were identified in the *ERG11* and *FKS2* genes, specifically the Y132F mutation in *ERG11* and the F635Y mutation in *FKS2*. These mutations are known to confer resistance to azole and echinocandin antifungal drugs. Interestingly, the same mutations were observed in all four isolates, suggesting a common mechanism of resistance. While mutations in *ERG11* and *FKS2* were detected, no mutations were detected in the *ERG3* and *FKS1* genes, indicating that mutations in *ERG11* and *FKS2* may be more prevalent or effective in conferring resistance in these isolates. All gene sequences were submitted to GenBank with the accession numbers listed in Table 3.

**Table 3:** Resistance genes and their mutation positions.

Sample No.	Gene	GenBank accession No.	Mutation	
			AA	NT
INSF1	*ERG3*	PP001723	-	-
INSF3		PP001724	-	-
INSF5		PP001725	-	-
INSF6		PP001726	-	-
INSF1	*ERG11*	OR987839	Y132F	T*A*C/T*T*C
INSF3		OR987840	Y132F	T*A*C/T*T*C
INSF5		OR987841	Y132F	T*A*C/T*T*C
INSF6		OR987842	Y132F	T*A*C/T*T*C
INSF1	*FKS1*	OR987843	-	-
INSF3		OR987844	-	-
INSF5		OR987845	-	-
INSF6		OR987846	-	-
INSF1	*FKS2*	OR987835	F635Y	T*G*C/T*A*C
INSF3		OR987836	F635Y	T*G*C/T*A*C
INSF5		OR987837	F635Y	T*G*C/T*A*C
INSF6		OR987838	F635Y	T*G*C/T*A*C

*AA: amino acid; NT: Nucleotide.

## Discussion

*Candida auris *infections add a huge burden on healthcare systems worldwide due to their severe impact, challenging diagnosis, and limited treatment options due to multidrug resistance^25–27^. The prevalence of *C. auris* infection has been extensively studied worldwide, there is a significant gap in our understanding of its prevalence and impact in Egypt. This study aimed to pinpoint the genetic determinants of antifungal resistance among *C. auris* isolates collected from healthcare facilities in Egypt.

Four *C. auris *strains were isolated from critically ill patients admitted to an Egyptian healthcare facility. All the test strains exhibited multidrug resistance to the first generation of azoles, echinocandins, and 5-flucytosine. The MIC values corresponded to those approved by CLSI as an indicator of fluconazole and caspofungin resistance^2,28,29^. Interestingly, it was reported that a minimum of 4% of *C. auris*infections are multidrug-resistant to the major classes of antifungal agents^30^.

Phylogenetic analysis was conducted to understand the evolutionary relationships and genetic diversity of *C. auris* strains. The ITS sequences were submitted to GenBank, contributing to the global database of *C. auris* genetic information. Previous studies on the phylogeny of *Candida* species showed that most of the pathogenic species belong to the CTG clade including *C. albicans*, *C. tropicalis*,* C. parapsilosis*, and *C. auris*^14^. *C. auris* belongs to the genus *Clavispora* (commonly referred to as *Candida*). The members of *Clavispora* are evolutionarily divergent from the *Lodderomyces* group species of *Candida*, which includes *C. albicans* and the other commonly encountered pathogenic *Candida *species^30^.

This contributes to a notably higher frequency of multidrug resistance among *Clavispora* clinical isolates. The haploid nature of *Clavispora *genomes allows a single recessive point mutation to be phenotypically expressed, leading to more frequent emergence of resistance^30^. A comprehensive examination of the antifungal resistance mechanisms in *C. auris*suggests that it can escape antifungal action through two primary mechanisms: altering the drug-target interaction or reducing the intracellular drug concentration. These alterations can be caused by modifications in the cell wall or efflux pumps or by changes in the target protein^14,30,31^.

To explore resistance mechanisms in *C. auris* test strains, four genes were selected for study: *ERG3*, *ERG11*, *FKS1*, and *FKS2*. Mutations in *ERG3* and *ERG11* were directly implicated in azole resistance, whereas mutations in *FKS1* and *FKS2 *were primarily associated with reduced susceptibility to echinocandins^13,30^.

Triazole antifungal agents (including fluconazole) exert their therapeutic effect by competitively inhibiting sterol demethylase, a vital enzyme for the biosynthesis of ergosterol in fungi^14,30,31^. Given the widespread *C. auris *isolates resistant to fluconazole, its efficacy against this pathogen is substantially reduced^32^. One frequently reported mechanism of fluconazole resistance in *C. auris* involves mutations within the *ERG11 *gene encoding the sterol-demethylase enzyme^28,33^. Three specific mutations have been repeatedly observed in resistant clinical isolates, involving the amino acid substitutions VF125AL, Y132F, and K143R^14^.

The results in the present study revealed exclusively Y132F mutations in the *ERG11* gene in all the tested strains with no mutations observed in the *ERG3* gene. This agrees with the literature where the Y132F mutation in *ERG11* is the most frequent mutation among *C. auris* strains and has been found in a wide range of *C. auris*isolates from various geographic regions and genetic clades^14,34^.

Interestingly, recent molecular studies suggest that *C. auris*displays clade-specific profiles of azole resistance^14,35^. The *ERG11 *Y132F mutation has been strongly associated with clades I and IV^14,35,41^. In contrast, The VF125AL mutation has been exclusively detected in isolates belonging to Clade III, while the K143R-encoding mutation has been reported solely in Clade-I isolates and a limited number of individual isolates from Clade II and Clade IV^14,36^.

Although the test strains were resistant to fluconazole, itraconazole, and ketoconazole, they were notably susceptible to both posaconazole and voriconazole. This was reported in other studies including *C. auris* isolates with mutations in the *ERG11*gene^16,37^. This differential resistance can be attributed to several factors. Generally, azoles are classified into two groups based on structural differences: first-generation azoles [imidazole (ketoconazole), triazoles (itraconazole, fluconazole)] and second-generation azoles (triazoles posaconazole and voriconazole)^38–41^. Both generations contain azole rings, but second-generation azoles often have more complex side chains and additional functional groups increasing their spectrum of activity and binding affinity to sterol demethylase even in the presence of *ERG11* mutations. On the other hand, *ERG11*mutations significantly reduce the binding affinity of fluconazole and itraconazole to the enzyme, leading to resistance^16,33,37^.

The test strains exhibited resistance to echinocandins, a class of antifungals that inhibit the synthesis of 1,3 -β-D-glucan, a crucial component of the fungal cell wall^42,43^. Mutations within the *FKS*genes, which encode the catalytic subunits of the 1,3 -β-D-glucan synthase enzyme, have been identified as the primary cause of echinocandin resistance^44^. These mutations result in amino acid substitutions within the *FKS* subunit of glucan synthase, significantly reducing the enzyme’s sensitivity to echinocandin. Generally, in *Candida* species, echinocandin resistance predominantly arises from the development of mutations in two highly conserved hot-spot regions of the *FKS* genes, which encode the catalytic subunits (*FKS* subunits) of β−1,3-D-glucan synthase^14,41,45^.

 Although both *FKS1* and *FKS2* genes encode subunits of the 1,3- β-D-glucan synthase enzyme, mutations within the *FKS1* gene represent the most frequently observed genetic alterations in *FKS* genes.* FKS2* mutations in echinocandin-resistant *C. auris* are relatively uncommon compared to mutations in the *FKS1 *gene^14,30,46^. Interestingly, all the test strains in the present study showed the less frequently encountered F635Y mutations in *FKS2* genes. Similar patterns were recorded for *C. glabrata *which expresses *FKS2* at higher levels than *FKS*1, and mutations in *FKS2 *may exert a more significant impact on echinocandin resistance in this species^45^.

It is clear from the previous results that the test strains might display an uncommon susceptibility profile. So, while the present data focuses on mutations in *ERG3*, *ERG11*, *FKS1*, and *FKS2*, Further studies on other mechanisms of resistance, such as overexpression of efflux pumps, may also be contributing to the antifungal resistance of these isolates. It is also important to analyze a larger number of isolates with whole genome assessment to understand the full spectrum of genetic variations and resistance mechanisms associated with *C. auris* in Egypt. To achieve this, it is crucial to broaden sample collection to include multiple centers, ensuring a diverse and representative dataset. Involving different multicenter settings will provide a more comprehensive understanding of the epidemiological and genomic landscape of *C. auris* in the region, enhancing the reliability and generalizability of the findings.

## Conclusion

*auris* has been causing serious infections in healthcare settings worldwide in the last decade, causing outbreaks among immunosuppressed individuals. Its resistance to multiple antifungal agents makes it challenging to treat, leading to increased morbidity and mortality. This study aims to provide key insights into the resistance patterns of *C. auris* and contribute to the general knowledge about this emerging pathogen in Egypt. The identification of these mutations adds valuable information for understanding the mechanisms of resistance, guiding targeted antifungal therapies, and developing effective control strategies.

## Electronic supplementary material

Below is the link to the electronic supplementary material.

Supplementary Material 1

Supplementary Material 2

## Data Availability

The data generated and analysed during the current study are available in the GenBank repository (https://www.ncbi.nlm.nih.gov/nuccore/) under the accession numbers OR976106-OR976107-OR976108-OR976109-PP001723-PP001724-PP001725-PP001726-OR987839-OR987840-OR987841-OR987842-OR987843-OR987844-OR987845-OR987846-OR987835-OR987836-OR987837-OR987838.

## Abbreviations

CDC: Centers for Disease Control & Prevention
WHO: World Health Organization
BAL: Bronchoalveolar lavage
MALDI-TOF: Matrix-assisted laser desorption ionization-time of flight mass spectrometry
PCR: Polymerase chain reaction
ITS: Internal transcribed spacer
NT: Nucleotide
AA: Amino acids
CLSI: Clinical and Laboratory Standards Institute
CTG: Candida clade
Flu: Fluconazole
Itra: Itraconazole
Keto: Ketoconazole
Fct: Flucytosine
Mcf: Micafungin
Caspo: Caspofungin
Pos: Posaconazole
Vori: Voriconazole
AB: Amphotericin B
S: Sensitive
R: Resistant
DM: Diabetes milites
