# Supplementary material for: Molecular characterization of some multidrug resistant Candida Auris in egypt
Source: Sci Rep. 2025 Feb 10;15:4917. doi: 10.1038/s41598-025-88656-3 (PMC11811120; doi:10.1038/s41598-025-88656-3)
Supplement: Supplementary file 1 — Supplementary Material 1 [file 41598_2025_88656_MOESM1_ESM.docx]

| **Name** | **Accession no.** | **GenBank link** |
| --- | --- | --- |
| INSF1 (ITS sequence) | OR976106 | <https://www.ncbi.nlm.nih.gov/nuccore/OR976106> |
| INSF3 (ITS sequence) | OR976107 | <https://www.ncbi.nlm.nih.gov/nuccore/OR976107> |
| INSF5 (ITS sequence) | OR976108 | <https://www.ncbi.nlm.nih.gov/nuccore/OR976108> |
| INSF6 (ITS sequence) | OR976109 | <https://www.ncbi.nlm.nih.gov/nuccore/OR976109> |
| INSF1 (ERG3) | PP001723 | <https://www.ncbi.nlm.nih.gov/nuccore/PP001723> |
| INSF3 (ERG3) | PP001724 | <https://www.ncbi.nlm.nih.gov/nuccore/PP001724> |
| INSF 5 (ERG3) | PP001725 | <https://www.ncbi.nlm.nih.gov/nuccore/PP001725> |
| INSF6 (ERG3) | PP001726 | <https://www.ncbi.nlm.nih.gov/nuccore/PP001726> |
| INSF1 (ERG11) | OR987839 | <https://www.ncbi.nlm.nih.gov/nuccore/OR987839> |
| INSF3 (ERG11) | OR987840 | <https://www.ncbi.nlm.nih.gov/nuccore/OR987840> |
| INSF 5 (ERG11) | OR987841 | <https://www.ncbi.nlm.nih.gov/nuccore/OR987841> |
| INSF6 (ERG11) | OR987842 | <https://www.ncbi.nlm.nih.gov/nuccore/OR987842> |
| INSF1 (FKS1) | OR987843 | <https://www.ncbi.nlm.nih.gov/nuccore/OR987843> |
| INSF3 (FKS1) | OR987844 | <https://www.ncbi.nlm.nih.gov/nuccore/OR987844> |
| INSF 5 (FKS1) | OR987845 | <https://www.ncbi.nlm.nih.gov/nuccore/OR987845> |
| INSF6 (FKS1) | OR987846 | <https://www.ncbi.nlm.nih.gov/nuccore/OR987846> |
| INSF1 (FKS2) | OR987835 | <https://www.ncbi.nlm.nih.gov/nuccore/OR987835> |
| INSF3 (FKS2) | OR987836 | <https://www.ncbi.nlm.nih.gov/nuccore/OR987836> |
| INSF 5 (FKS2) | OR987837 | <https://www.ncbi.nlm.nih.gov/nuccore/OR987837> |
| INSF6 (FKS2) | OR987838 | <https://www.ncbi.nlm.nih.gov/nuccore/OR987838> |
