# Supplementary material for: Molecular characterization of some multidrug resistant Candida Auris in egypt
Source: Sci Rep. 2025 Feb 10;15:4917. doi: 10.1038/s41598-025-88656-3 (PMC11811120; doi:10.1038/s41598-025-88656-3)
Supplement: Supplementary file 2 — Supplementary Material 2 [file 41598_2025_88656_MOESM2_ESM.docx]

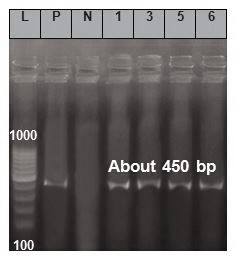


**Fig 1.** Gel electrophoresis of the amplified ITS sequences. **L**: DNA Ladder (100 bp and 1000 bp); **P**: positive control; **N**: negative control; **1:** INSF1; **2:** INSF3; **3:** INSF5; and **4:** INSF6.


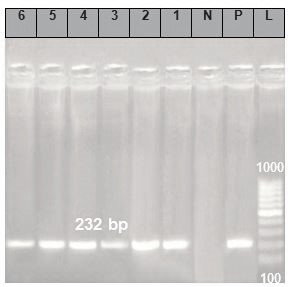

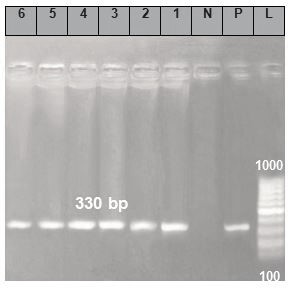

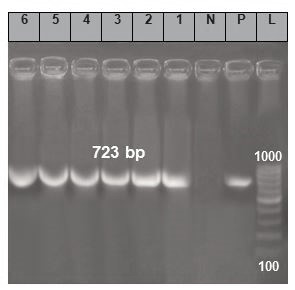

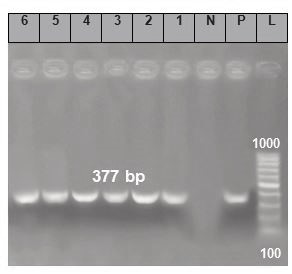


**A**

**B**

**D**

**C**

**Fig 3.** Gel electrophoresis of the amplified genes: **(a)** *ERG3*; (b) *ERG11*; (c) *FKS1*; (d) *FKS2*; **L**: DNA Ladder (100 bp and 1000 bp); **P**: positive control; **N**: negative control; **1:** INSF1; **2:** INSF3; **3:** INSF5; and **4:** INSF6

**Note: We cropped the last two lanes in the gel photos in figure numbers 3a-3d in the main manuscript because they were duplicates for samples number 3&4.**
